# Poly[[μ-1,4-bis­(4,5-dihydro-1,3-oxazol-2-yl)benzene-κ^2^ *N*:*N*′]di-μ-bromido-cadmium]

**DOI:** 10.1107/S1600536811027759

**Published:** 2011-07-16

**Authors:** Maw-Cherng Suen, Chun-Wei Yeh, Shui-Chuan Lin, Yi-Fen Hsu

**Affiliations:** a Department of Material and Fiber, Nanya Institute of Technology, Chung-Li, Taiwan; b Department of Chemistry, Chung-Yuan Christian University, Chung-Li, Taiwan; c Department of Polymer Materials, Vanung University, Jhongli, Taiwan

## Abstract

In the title coordination polymer, [CdBr_2_(C_12_H_12_N_2_O_2_)]_*n*_, the Cd^II^ ion, situated on an inversion centre, is coordinated by four bridging Br atoms and two N atoms from two 1,4-bis­(4,5-dihydro-1,3-oxazol-2-yl)benzene (*L*) ligands in a distorted octa­hedral geometry. The *L* ligand, which also lies across an inversion centre, bridges two Cd^II^ ions, forming layers parallel to (010).

## Related literature

For background to coordination polymers with organic ligands, see: Chiang *et al.* (2008 ▶); Hsu *et al.* (2009 ▶); Kitagawa *et al.* (2004 ▶); Yeh *et al.* (2008 ▶, 2009 ▶). For Cd(II) coordination polymers, see: Suen & Wang (2007*a* ▶,*b* ▶). For related structures, see: Wang *et al.* (2008 ▶, 2011 ▶).
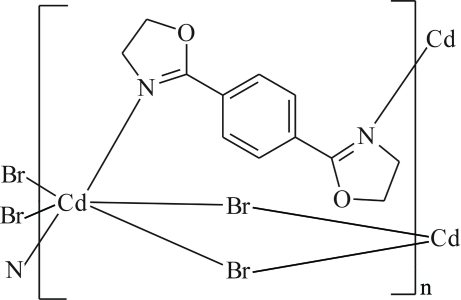


## Experimental

### 

#### Crystal data

[CdBr_2_(C_12_H_12_N_2_O_2_)]
*M* *_r_* = 488.46
Triclinic, 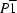

*a* = 4.0595 (2) Å
*b* = 8.1114 (3) Å
*c* = 10.1132 (4) Å
α = 84.503 (2)°
β = 81.963 (2)°
γ = 84.898 (2)°
*V* = 327.26 (2) Å^3^
*Z* = 1
Mo *K*α radiation
μ = 7.77 mm^−1^
*T* = 296 K
0.16 × 0.06 × 0.06 mm

#### Data collection

Bruker APEXII CCD diffractometer
Absorption correction: multi-scan (*SADABS*; Bruker, 2001 ▶) *T*_min_ = 0.769, *T*_max_ = 0.971
15213 measured reflections
4234 independent reflections
3127 reflections with *I* > 2σ(*I*)
*R*_int_ = 0.081

#### Refinement

*R*[*F* ^2^ > 2σ(*F* ^2^)] = 0.026
*wR*(*F* ^2^) = 0.067
*S* = 0.96
4234 reflections
88 parameters
H-atom parameters constrained
Δρ_max_ = 0.99 e Å^−3^
Δρ_min_ = −1.55 e Å^−3^

### 

Data collection: *APEX2* (Bruker, 2007 ▶); cell refinement: *SAINT* (Bruker, 2007 ▶); data reduction: *SAINT*; program(s) used to solve structure: *SHELXS97* (Sheldrick, 2008 ▶); program(s) used to refine structure: *SHELXL97* (Sheldrick, 2008 ▶); molecular graphics: *DIAMOND* (Brandenburg, 1999 ▶); software used to prepare material for publication: *SHELXL97*.

## Supplementary Material

Crystal structure: contains datablock(s) I, global. DOI: 10.1107/S1600536811027759/hy2448sup1.cif

Structure factors: contains datablock(s) I. DOI: 10.1107/S1600536811027759/hy2448Isup2.hkl

Additional supplementary materials:  crystallographic information; 3D view; checkCIF report

## Figures and Tables

**Table 1:** Selected bond lengths (Å)

Cd—N	2.5189 (12)
Cd—Br	2.7085 (2)
Cd—Br^i^	2.7901 (2)

Symmetry code: (i) 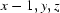.

**Fig. 1.**
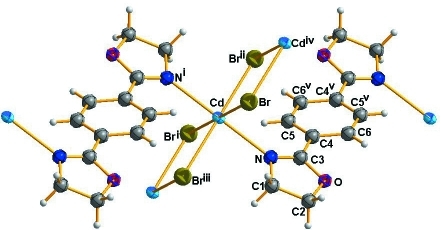
A portion of the two-dimensional network in the title compound. Displacement ellipsoids are drawn at the 30% probability level. [Symmetry codes: (i) -x + 1, -y + 1, -z; (ii) -x, -y + 1, -z; (iii) x + 1, y, z; (iv) x - 1, y, z; (v) -x, -y + 1, -z + 1.]

**Fig. 2.**
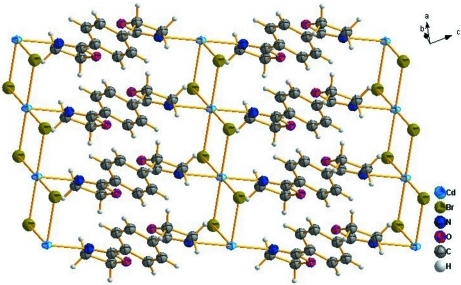
A drawing of the two-dimensional network.

## supplementary crystallographic information

### Comment

The synthesis of metal coordination polymers has been a subject of intense research due to their interesting structural chemistry and potential applications in gas storage, separation, catalysis, magnetism, luminescence, and drug delivery (Kitagawa *et al.*, 2004). Roles of anions, solvents and ligand conformations in the self-assembly of coordination complexes containing polydentate nitrogen ligands are very interesting (Chiang *et al.*, 2008; Hsu *et al.*, 2009; Yeh *et al.*, 2008, 2009). The Cd(II) complexes containing polydentate ligands showing various types of frameworks are also reported (Suen & Wang, 2007*a*,b). The Ag(I) and Cu(II) complexes containing 1,4-bis(4,5-dihydro-2-oxazolyl)benzene (*L*) ligands have been reported, which show various one- and two-dimensional networks (Wang, Lee *et al.*, 2008; Wang, Yeh *et al.*, 2011).

In the title complex, the Cd^II^ ion is six-coordinated with four Br atoms and two N atoms from two *L* ligands (Fig. 1, Table 1). The Cd···Cd distances separated by the bridging *L* ligands and Br atoms are 10.3574 (4) and 4.0595 (2) Å. The ligand adopts an *anti* conformation in the structure (Fig. 2).

### Experimental

An aqueous solution (5.0 ml) of cadmium bromide (1.0 mmol) was layered carefully over a methanolic solution (5.0 ml) of 1,4-bis(4,5-dihydro-2-oxazolyl)benzene (1.0 mmol) in a tube. Colourless crystals were obtained after several weeks. These were washed with methanol and collected in 69.8% yield.

### Refinement

H atoms were positioned geometrically and refined as riding atoms, with C—H = 0.93 (phenyl) and 0.97 (methylene) Å and *U*_iso_(H) = 1.2*U*_eq_(C).

### Crystal data

**Table:**

[CdBr_2_(C_12_H_12_N_2_O_2_)]	*Z* = 1
*M**_r_* = 488.46	*F*(000) = 232
Triclinic, *P*1	*D*_x_ = 2.478 Mg m^−^^3^
Hall symbol: -P 1	Mo *K*α radiation, λ = 0.71073 Å
*a* = 4.0595 (2) Å	Cell parameters from 7163 reflections
*b* = 8.1114 (3) Å	θ = 3.1–41.1°
*c* = 10.1132 (4) Å	µ = 7.77 mm^−^^1^
α = 84.503 (2)°	*T* = 296 K
β = 81.963 (2)°	Columnar, colourless
γ = 84.898 (2)°	0.16 × 0.06 × 0.06 mm
*V* = 327.26 (2) Å^3^	

### Data collection

**Table:**

Bruker APEXII CCD diffractometer	4234 independent reflections
Radiation source: fine-focus sealed tube	3127 reflections with *I* > 2σ(*I*)
graphite	*R*_int_ = 0.081
φ and ω scans	θ_max_ = 41.1°, θ_min_ = 3.1°
Absorption correction: multi-scan (*SADABS*; Bruker, 2001)	*h* = −7→6
*T*_min_ = 0.769, *T*_max_ = 0.971	*k* = −14→14
15213 measured reflections	*l* = −18→18

### Refinement

**Table:**

Refinement on *F*^2^	Primary atom site location: structure-invariant direct methods
Least-squares matrix: full	Secondary atom site location: difference Fourier map
*R*[*F*^2^ > 2σ(*F*^2^)] = 0.026	Hydrogen site location: inferred from neighbouring sites
*wR*(*F*^2^) = 0.067	H-atom parameters constrained
*S* = 0.96	*w* = 1/[σ^2^(*F*_o_^2^) + (0.0224*P*)^2^] where *P* = (*F*_o_^2^ + 2*F*_c_^2^)/3
4234 reflections	(Δ/σ)_max_ < 0.001
88 parameters	Δρ_max_ = 0.99 e Å^−^^3^
0 restraints	Δρ_min_ = −1.55 e Å^−^^3^

### Fractional atomic coordinates and isotropic or equivalent isotropic displacement parameters (Å^2^)

**Table:**

	*x*	*y*	*z*	*U*_iso_*/*U*_eq_	
Cd	0.5000	0.0000	0.0000	0.02232 (4)	
N	0.4635 (3)	−0.21212 (15)	0.19936 (11)	0.0222 (2)	
O	0.2887 (3)	−0.39410 (14)	0.37175 (11)	0.0361 (3)	
C1	0.5970 (4)	−0.37792 (18)	0.15877 (14)	0.0282 (3)	
H1B	0.8389	−0.3851	0.1436	0.034*	
H1A	0.5149	−0.4007	0.0773	0.034*	
C2	0.4731 (5)	−0.4991 (2)	0.27480 (16)	0.0333 (3)	
H2B	0.3300	−0.5751	0.2468	0.040*	
H2A	0.6583	−0.5627	0.3115	0.040*	
C3	0.3062 (4)	−0.23541 (17)	0.31777 (13)	0.0212 (2)	
C4	0.1447 (3)	−0.11180 (17)	0.40873 (12)	0.0200 (2)	
C5	0.2347 (4)	0.05114 (18)	0.39301 (13)	0.0227 (2)	
H5	0.3917	0.0853	0.3219	0.027*	
C6	−0.0891 (4)	−0.16227 (18)	0.51607 (13)	0.0229 (2)	
H6	−0.1479	−0.2714	0.5267	0.027*	
Br	0.91841 (3)	0.179762 (17)	0.103470 (13)	0.02270 (4)	

### Atomic displacement parameters (Å^2^)

**Table:**

	*U*^11^	*U*^22^	*U*^33^	*U*^12^	*U*^13^	*U*^23^
Cd	0.01836 (6)	0.02238 (7)	0.02671 (7)	−0.00089 (5)	−0.00254 (4)	−0.00583 (5)
N	0.0272 (5)	0.0202 (5)	0.0181 (5)	−0.0007 (4)	0.0012 (4)	−0.0038 (4)
O	0.0540 (8)	0.0203 (5)	0.0268 (5)	0.0041 (5)	0.0134 (5)	0.0000 (4)
C1	0.0369 (8)	0.0205 (6)	0.0237 (6)	0.0029 (5)	0.0056 (5)	−0.0034 (5)
C2	0.0432 (9)	0.0211 (7)	0.0304 (7)	0.0038 (6)	0.0093 (6)	−0.0029 (6)
C3	0.0253 (6)	0.0189 (6)	0.0187 (5)	−0.0003 (5)	−0.0008 (4)	−0.0026 (4)
C4	0.0234 (6)	0.0216 (6)	0.0149 (5)	0.0007 (5)	−0.0018 (4)	−0.0036 (4)
C5	0.0269 (6)	0.0241 (6)	0.0160 (5)	−0.0025 (5)	0.0020 (4)	−0.0019 (4)
C6	0.0288 (6)	0.0205 (6)	0.0188 (5)	−0.0031 (5)	0.0005 (4)	−0.0032 (4)
Br	0.02070 (7)	0.02520 (7)	0.02242 (7)	−0.00113 (5)	−0.00048 (4)	−0.00718 (5)

### Geometric parameters (Å, °)

**Table:**

Cd—N	2.5189 (12)	C2—H2B	0.9700
Cd—Br	2.70850 (16)	C2—H2A	0.9700
Cd—Br^i^	2.79006 (16)	C3—C4	1.4739 (17)
N—C3	1.2813 (17)	C4—C5	1.3910 (19)
N—C1	1.4781 (18)	C4—C6	1.3940 (19)
O—C3	1.3530 (18)	C5—C6^ii^	1.3855 (18)
O—C2	1.4451 (17)	C5—H5	0.9300
C1—C2	1.516 (2)	C6—C5^ii^	1.3855 (18)
C1—H1B	0.9700	C6—H6	0.9300
C1—H1A	0.9700		
			
N—Cd—N^iii^	180.00 (6)	N—C1—H1A	110.7
N—Cd—Br^iii^	86.94 (3)	C2—C1—H1A	110.7
N^iii^—Cd—Br^iii^	93.06 (3)	H1B—C1—H1A	108.8
N—Cd—Br	93.06 (3)	O—C2—C1	103.97 (11)
N^iii^—Cd—Br	86.94 (3)	O—C2—H2B	111.0
Br^iii^—Cd—Br	180.000 (5)	C1—C2—H2B	111.0
N—Cd—Br^iv^	87.67 (3)	O—C2—H2A	111.0
N^iii^—Cd—Br^iv^	92.33 (3)	C1—C2—H2A	111.0
Br^iii^—Cd—Br^iv^	95.159 (5)	H2B—C2—H2A	109.0
Br—Cd—Br^iv^	84.841 (5)	N—C3—O	117.42 (12)
N—Cd—Br^i^	92.33 (3)	N—C3—C4	129.11 (13)
N^iii^—Cd—Br^i^	87.67 (3)	O—C3—C4	113.44 (11)
Br^iii^—Cd—Br^i^	84.841 (5)	C5—C4—C6	119.85 (12)
Br—Cd—Br^i^	95.159 (5)	C5—C4—C3	121.06 (11)
Br^iv^—Cd—Br^i^	180.000 (6)	C6—C4—C3	119.00 (12)
C3—N—C1	106.43 (12)	C6^ii^—C5—C4	119.60 (12)
C3—N—Cd	140.65 (10)	C6^ii^—C5—H5	120.2
C1—N—Cd	110.60 (8)	C4—C5—H5	120.2
C3—O—C2	106.97 (11)	C5^ii^—C6—C4	120.55 (13)
N—C1—C2	105.15 (11)	C5^ii^—C6—H6	119.7
N—C1—H1B	110.7	C4—C6—H6	119.7
C2—C1—H1B	110.7	Cd—Br—Cd^v^	95.159 (5)

Symmetry codes: (i) *x*−1, *y*, *z*; (ii) −*x*, −*y*, −*z*+1; (iii) −*x*+1, −*y*, −*z*; (iv) −*x*+2, −*y*, −*z*; (v) *x*+1, *y*, *z*.
